# Down-regulation of cathepsin S and matrix metalloproteinase-9 via Src, a non-receptor tyrosine kinase, suppresses triple-negative breast cancer growth and metastasis

**DOI:** 10.1038/s12276-018-0135-9

**Published:** 2018-09-05

**Authors:** Jaya Gautam, Suhrid Banskota, Hyunji Lee, Yu-Jeong Lee, Yong Hyun Jeon, Jung-Ae Kim, Byeong-Seon Jeong

**Affiliations:** 1 College of Pharmacy, Yeungnam University, Gyeongsan, 38541 Republic of Korea; 2 Department of Nuclear Medicine, Kyungpook National University, Daegu, 41944 Republic of Korea

## Abstract

Triple-negative breast cancer (TNBC) is a highly metastatic breast cancer with poor prognosis. In the present study, we demonstrated that Src, a non-receptor tyrosine kinase, might provide an effective therapeutic strategy to overcome TNBC invasion and metastasis, which are mediated via the synergistic action of the lysosomal enzyme cathepsin S (CTSS) and gelatinase MMP-9. Knock-down of MMP-9 and CTSS using siRNAs resulted in a synergistic suppression of MDA-MB-231 cell invasion, which was similarly observed with pharmacological inhibitors. During the screening of new drug candidates that suppress both CTSS and MMP-9, BJ-2302, a novel 7-azaindolin-2-one derivative, was discovered. Src, an upstream activator of both pathways (PI3K/Akt and Ras/Raf/ERK) responsible for the expression of CTSS and MMP-9, was identified as a high-affinity target of BJ-2302 (IC_90_: 3.23 µM) through a Src kinase assay and a drug affinity responsive target stability (DARTS) assay. BJ-2302 effectively suppressed MDA-MB-231 cell invasion (Matrigel invasion assay) and metastasis (chorioallantoic membrane assay xenografted with MDA-MB-231-luc2-tdTomato cancer cells). Unlike Z-FL-COCHO (potent CTSS inhibitor), BJ-2302 did not induce any cytotoxicity in MCF-10A normal breast epithelial cells. Additionally, BJ-2302 (1 mg/kg) strongly suppressed TNBC cell proliferation in vitro and tumor growth in a xenograft mouse tumor model. The anti-metastatic and anti-tumor effects of BJ-2302 were superior to those of Z-FL-COCHO (1 mg/kg) or batimastat (30 mg/kg), a pan-MMP inhibitor. In summary, inhibition of Src kinase suppressed TNBC tumor growth and metastasis, and Src inhibitors such as BJ-2302 may constitute a novel therapeutic tool to treat breast cancer that expresses high levels of CTSS and MMP-9.

## Breast cancer: Trapping tumors in place

Drugs that inhibit two protein-digesting enzymes could offer potent protection against tumor growth and metastasis in an aggressive form of breast cancer. “Triple-negative breast cancer” (TNBC), which lacks three common tumor biomarkers, carries a poor prognosis for patients, with few treatment options. Enzymes that degrade the protein matrix that anchors tumor cells play a prominent role in metastasis. Researchers led by Jung Ae Kim and Byeong-Seon Jeong at Yeungnam University, Gyeongsan, South Korea, have demonstrated that these enzymes offer potential therapeutic exploitation. The researchers identified a compound that simultaneously suppresses the activity of two different matrix-digesting enzymes. This parallel inhibition markedly reduced tumor growth and metastatic invasion in mouse models of TNBC, with minimal toxicity to non-cancerous cells. This approach could thus offer new hope for treating a challenging class of tumors.

## Introduction

Triple-negative breast cancer (TNBC) that does not express estrogen receptor (ER), progesterone receptor (PR), and HER-2/neu is the most aggressive sub-type of breast cancer. Although the survival rate of breast cancer patients has been increased by molecular targeted therapies against HER2 or hormone receptors^1–3^, TNBC is associated with the worst prognosis^4^, and TNBC patients show relapse and frequently develop metastasis in visceral organs.

During the process of invasion and metastasis, although the invading cancer cells confront numerous tissue barriers (basement membranes and interstitial connective tissues), these membranes are degraded by various types of proteases that are released from invading cancer cells or stromal cells that interact with cancer cells. Among various metalloproteases, gelatinase matrix metalloprotease (MMP)-9 has been reported as a potentially useful biomarker for the aggressive subtype of breast cancer^5^. Additionally, elevated tissue levels of MMP-9 were found to be associated with triple-negativity^5^, poor prognosis^6^, regional node metastases, shorter time to relapse, and reduced survival after relapse^5^ in breast cancer patients. For many years, the importance of other proteases has been overshadowed by the matrix MMPs in the field of cancer metastasis. However, the clinical failure of MMP inhibitors to prevent cancer metastasis led to the investigation of other proteases such as cathepsins as promising candidates for use in the management of cancer metastasis. Different members of the cathepsin family have been an intense focus in the cancer field based on their increased expression and activity compared to their normal counterparts^7^. Cathepsins are localized primarily in lysosomes. However, they are secreted on the cell surface in the highly acidic tumor microenvironment, which leads to the degradation of various extracellular matrix (ECM) proteins and basement membranes and results in the promotion of tumor cell invasion and metastasis^8,9^. Previous studies have demonstrated the key role of different types of cathepsins in cancer cell invasion, such as cathepsin (CTS) B in glioma^10^, CTSL in ovarian carcinoma^11^, and CTSS in colorectal^12^ and breast cancer metastasis^13^. Recently, elevated levels of CTSS in triple-negative breast tumor tissues have been demonstrated to play a critical role in MDA-MB-231 TNBC invasion^14^. In addition, CTSS has been suggested as a therapeutic target that does not induce any detrimental effects on MHC class II function, not only in cancer but also in the management of autoimmunity-related tissue-degrading diseases such as arthritis^15^. CTSS has been reported to have limited tissue distribution^16^, and such special attributes make CTSS a good target for the management of cancer metastasis. A number of cathepsins have been found to activate MMP-9 through the proteolysis of its pro-domain^17,18^. Although complementary roles of CTSB and MMP-9 have been demonstrated in glioblastoma^19^ and prostate carcinoma^20^, such a cooperative role of CTSB or CTSS with MMP-9 has not yet been revealed in breast cancer progression.

A previous study has shown that CTSS expression in TNBC is regulated via dual signaling pathways, namely, PI3K/Akt and Ras/Raf/ERK^14^, leading to the activation of NF-κB, which is also a regulator of MMP-9. In addition, the PI3K/Akt and Ras/Raf/ERK pathways in TNBC cells are controlled by Src upon activation of both growth factor receptors (GFRs) and 5-HT_7_ receptor^21^. Clinically, Src activity is increased in invasive breast tumor tissues compared with normal tissue^22–24^, and the action of Src kinase in integrating both PI3K/Akt and Ras/Raf/ERK signaling pathways^25^ in cancer progression has made it a potential target for cancer therapeutics. In conjunction with reports that TNBC cells are highly sensitive to Src-targeting small-molecule inhibitors^26,27^, Src has been suggested as a novel therapeutic target to treat basal breast cancer and TNBC^28,29^. During the development of anti-cancer drugs that target Src, the efficacy of the inhibitors was found to be lacking during phase II clinical trials. However, a critical factor that contributed to such unsuccessful results was the lack of biomarkers to identify the patients most likely to respond to such therapy^30,31^. Similar to endocrine therapy or anti-HER2 therapy, which requires determination of ER/PR or HER2 expression in breast cancer, respectively, it is necessary to identify biomarkers in TNBC to correlate the outcome of anti-Src therapy.

Taking these findings into consideration, in the present study, we investigated the regulatory role of Src in TNBC progression in a stepwise approach: (1) determining if both CTSS and MMP-9 levels are highly increased in TNBC; (2) determining if dual inhibition of CTSS and MMP-9 synergistically suppresses TNBC invasion and metastasis; (3) identifying a compound that inhibits both CTSS and MMP-9; and (4) determining if the effect of the compound is mediated through Src inhibition.

## Materials and methods

### Materials

DMEM high glucose, fetal bovine serum (FBS), M-PER buffer, and penicillin and streptomycin antibiotics were purchased from Thermo Scientific HyClone (Logan, UT, USA). 3-(4,5-Dimethylthiazol-2-yl)-2,5-diphenyltetrazolium bromide (MTT) was obtained from Sigma-Aldrich (St. Louis, MO, USA). Matrigel was obtained from BD Biosciences (Bedford, MA, USA). Hematoxylin was purchased from Scytek Laboratories (Logan, UT, USA). CA-074 Me ([(2S, 3S)-3-propylcarbamoyloxirane-2-carbonyl]-L-isoleucyl-L-prolinemethyl ester) was purchased from Peptide Institute, Inc. (Ina Minoh-shi, Osaka, Japan). Pepstatin A (Isovaleryl-Val-Val-Sta-Ala-Sta) was obtained from Santa Cruz Biotechnology, Inc. (Delaware Ave, Santa Cruz, CA). Odanacatib ((2S)-N-(1-cyanocyclopropyl)-4-fluoro-4-methyl-2-{[(1S)-2,2,2-trifluoro-1-{4′-(methylsulfonyl)[1,1′-biphenyl]-4-yl}ethyl]amino}-pentanamide) was purchased from Selleck Chemicals (Houston, TX, USA). Z-FF-FMK and Z-FL-COCHO were obtained from EMD Chemicals, Inc. (San Diego, CA). Pronase was obtained from Roche Diagnostics (Roche, IN, USA). Antibodies directed against Src, phospho-p85-PI3K (at Tyr488), p85-PI3K, phospho-AKT (at T308), AKT, phospho-ERK, ERK, phospho-p38, p38, NF-κB, p-IκB, and IκB were purchased from Cell Signaling Technology Inc. (Beverly, MA, USA). β-Actin, CTSS, Lamin B, p-Ras, and Raf antibodies were purchased from Santa Cruz Biotechnology (Santa Cruz, CA, USA). MMP-9 and MMP-2 antibodies were obtained from Abcam (Cambridge, MA, USA), and 5-HT_7_ antibody was obtained from Novus Biologicals (Novus Biologicals, Littleton, CO).

### Cell culture

MDA-MB-231 and MCF-7 human breast cancer cells were obtained from American Type Culture Collection (ATCC, Manassas, VA, USA). HCC-1395, Hs578T, T47D, and MCF-10A cells were purchased from Korean Cell Line Bank (Seoul, Korea). MDA-MB-231, MCF-7, and Hs578T cells were cultured in DMEM high glucose supplemented with 10% FBS, 100 IU/ml penicillin, and 100 μg/ml streptomycin. HCC-1395, T47D, and MCF-10A cells were grown in RPMI1640 medium with 10% FBS, 100 IU/ml penicillin, and 100 μg/ml streptomycin. The cells were continuously maintained at 37 °C in a 5% CO_2_-humidified incubator.

### Real-time polymerase chain reaction (q-PCR)

Total RNA from MDA-MB-231 cells and tumor tissues was isolated using Trizol reagent (Life Technologies Inc., Grand Island, NY). Next, the extracted RNA was reverse transcribed into cDNA using a GoScript Reverse Transcription system (Promega Corporation, WI, USA). Quantitative analysis of mRNA was performed in the Corbett Rotor-Gene using a mixture of QuantiTect SYBR Green (Qiagen, CA, USA) and primer sequences, which are summarized in Table 1 (Corbett Life Science). *GAPDH* was used as a loading control.

**Table 1:** Primer sequences used in the qRT-PCR experiments

Gene	Human
CTSB (F)	5′-GATCTGCATCCACACCAATG-3′
CTSB (R)	5′-AACCAGGCCTTTTCTTGTCC-3′
CTSD (F)	5′-GACACAGGCACTTCCCTCAT-3′
CTSD (R)	5′-CTCTGGGGACAGCTTGTAGC-3′
CTSK (F)	5′-TTCTGCTGCTACCTGTGGTG-3′
CTSK (R)	5′-GCCTCAAGGTTATGGATGGA-3′
CTSL (F)	5′-CAGTGTGGTTCTTGTTGGGCT-3′
CTSL (R)	5′-CTTGAGGCCCAGAGCAGTCTA-3′
CTSS (F)	5′-GACACAGGCACTTCCCTCAT-3′
CTSS (R)	5′-GTTGTGGCCCCAGCTGTT-3′
MMP-2 (F)	5′-TGGCAAGTACGGCTTCTGTC-3′
MMP-2 (R)	5′-TTCTTGTCGCGGTCGTAGTC-3′
MMP-9 (F)	5′-GCTCACCTTCACTCGCGTG-3′
MMP-9 (R)	5′-CGCGACACCAAACTGGATG-3′
HPRT (F)	5′-TTCCTTGGTCAGGCAGCGTG-3′
HPRT (R)	5′-CGCGACACCAAACTGGATG-3′

*F* forward, *R* reverse

### Protein extraction and western blotting

Whole cell lysates were prepared using a radioimmunoprecipitation assay (RIPA) buffer containing 1× protease and phosphatase inhibitor cocktail (Thermo Scientific, CA, USA). RIPA buffer was composed of 150 mM sodium chloride, 1% Triton X-100, 0.5% sodium deoxycholate, 0.1% SDS, and 50 mM Tris adjusted to pH 8.0. After lysing cells with RIPA buffer, the cells were centrifuged at 16,200×*g* for 15 min, and supernatants containing soluble proteins were collected. Cytoplasmic and nuclear proteins were extracted from cells using an NE-PER kit (Pierce-Thermo, Logan, UT, USA).

Protein concentrations were measured using the BCA protein assay reagent (Pierce, Rockford, IL, USA). Equal amounts of total proteins were then separated through SDS-PAGE and were transferred onto Hybond ECL nitrocellulose membranes (Amersham Life Science, Buckinghamshire, UK) at 200 mA for an hour. The membranes were blocked in 5% skim milk in Tris-buffered saline (TBS)-Tween 20 (TBS-T) at room temperature (20–23 °C) for 1 h, incubated in skim milk-TBS overnight at 4 °C, washed three times with TBS-T, and incubated with horseradish peroxidase-conjugated secondary antibody in skim milk-TBS for 1 h at room temperature. Immunoreactive proteins were visualized using an enhanced chemiluminescence (ECL) kit (Pierce, Rockford, IL, USA) and were digitally processed using an LAS-4000 mini (Fuji, Japan). The membranes were stripped and reprobed with an actin antibody as a loading control. Densitometric analysis of the blots was performed using the Multi Gauge Ver 3.2 imaging software in a Fuji Image Station.

### siRNA transfection assay

MDA-MB-231 cells were seeded in antibiotic-free DMEM high glucose. When the cells were 60–70% confluent, the cells were transfected with the indicated siRNAs using the transfection reagent Dharmafect 4 purchased from Dharmacon (Lafayette, CO, USA). After 24 h of transfection, the cells were trypsinized and seeded for the cell viability and invasion assays (as described below).

### Cell viability measurement

MDA-MB-231 and MCF-10A cells cultured in a growth media were seeded in 96-well plates at a seeding density of 2 × 10^4^ cells/well. After 24 h, the media was replaced with serum-free media. The cells were treated with CTS inhibitors and CTS siRNAs at the indicated concentrations for 24 h. After 24 h of treatment, the medium was removed, and 20 µl of 3-MTT dye solution was added. After 4 h, the solution was removed and replaced with 200 µl of DMSO for 30 min at 37 °C. The absorbance was measured at 450 nm using a UV spectrophotometer.

### Matrigel invasion assay

The cell invasion assay was evaluated using Transwell inserts containing an 8.0-μm pore size membrane (Corning, NY, USA). Briefly, the inner surface of the membrane was coated with 20 µl of Matrigel (0.5 mg/ml) and the outer surface with 30 µl of type I collagen (0.5 mg/ml). After drying, the MDA-MB-231 cells were trypsinized and seeded at a density of 5 × 10^4^ cells/100 µl in the upper chamber. Media with or without 5% FBS was added to the bottom chamber. After 18 h of incubation at 37 °C, the invaded cells were fixed with methanol, stained with Mayer’s Hematoxylin and Eosin, and the stained cells were counted using a microscope at 200× magnification.

### Cathepsin activity measurement

CTSS and CTSB activities were measured using a fluorometric screening kit for CTSB (#K147, BioVision Inc., CA, USA) and for CTSS (#K149, BioVision Inc., CA, USA) according to the manufacturer’s protocol and were analyzed using a FLUOstar OPTIMA microplate reader (BMG Labtech GmbH, Offenburg, Germany) in fluorescence mode. The principle relies on the ability of cathepsins to cleave the synthetic AFC-based peptide substrate to release AFC, which can be measured using a fluorometer.

### Metastasis assay using CAM (chick chorioallantoic membrane)

Fertile chicken eggs purchased from Sib Rigol Farm (Daegu, Korea) were incubated at 37 °C and under 55% relative humidity. On the 11th day of egg incubation, using a grinding wheel (Dremel, Racine, WI, USA), a small window (1 cm^2^) was created on the superior surface of the eggs by separating the shell and membrane beneath. Next, MDA-MB-231 luc2-tdTomato breast cancer cells, which emit red fluorescence signal and allow for easy detection of migrating and metastasizing cancer cells, were loaded at a density of 2 × 10^6^ cells/CAM with or without Z-FL-COCHO (1 μM), batimastat (10 μM), and BJ-2302 (0.1 and 1 μM). After 5 days of drug treatment, the tumor weight, number of vessel branch points within the tumor region, and cell metastasis in the lungs and liver of the developing chicken embryo were analyzed.

The chick embryo experiments were performed following the institutional guidelines of the Institute of Laboratory Animal Resources (1996) and Yeungnam University for the care and use of laboratory animals (2009).

### Src kinase assay

Src kinase inhibitory activity of BJ-2302 was performed following the manufacturer’s protocol for the Cyclex c-Src Kinase Assay/Inhibitor Screening Kit (#CY-1083). The reaction was initiated with the addition of 10 µl of the recombinant catalytic domain of c-Src, 80 µl of kinase reaction buffer, and 10 µl of BJ-2302 (final concentration of 0.01–1 µM) in 96-well plates pre-coated with tyrosine kinase-substrate-1. The activity was measured colorimetrically at 450 nm using a microplate reader (Versamax, Molecular Devices, Sunnyvale, CA, USA).

### Cell proliferation assay

MDA-MB-231 cells were seeded at a density of 5000 cells/well in a 96-well plate. After overnight incubation, the cells were serum-starved using 0.2% FBS. The next day, the cells were pre-treated with the indicated concentrations of drugs for 1 h prior to the treatment with serum (5% FBS) or 5-HT (10 µM). After 72 h of incubation, MTT dye solution was added and incubated for 4 h. Next, DMSO was added, and after 30 min, the color intensities were measured using a microplate reader (Versamax, Molecular Devices, Inc., USA) at 490 nm.

### Drug affinity responsive target stability (DARTS) assay

The DARTS assay was performed following a previously described method^32^. Briefly, MDA-MB-231 cells (3 × 10^7^) were lysed with M-PER lysis buffer for 10 min, and the cell lysates were centrifuged at 18,000×*g* for 15 min. Next, the collected supernatants were mixed with 10× TNC buffer (500 mM Tris–HCl (pH 8.0), 500 mM NaCl, 100 mM CaCl_2_) and were incubated with or without BJ-2302 at the indicated concentrations for 1 h. After drug incubation, the proteins were digested with Pronase (at the indicated protease to protein ratios) for 30 min. The protein digestion was stopped by adding 4× sample buffer and heating the samples to 95 °C for 5 min. The samples were then separated via SDS-PAGE and were analyzed via western blotting.

### Animal experiments

Seven-to-eight weeks old female BALB/C nude mice were obtained from Orient Co. Ltd. (Seoul, Korea). The mice were maintained at 22 ± 1°C with a 12 h dark–light cycle with access to food and water. Cages for the mice were sterilized and kept in a clean room. MDA-MB-231-effLuc cells (1 × 10^7^ cells) in a Matrigel (with the ratio 1:1) were injected subcutaneously into the right flank of the mice. After reaching a tumor size of 200 mm^3^, the mice were administered vehicle or the indicated doses of Z-FL-COCHO, batimastat, or BJ-2302 intraperitoneally (i.p.) daily. After 25 days of drug treatment, the mice were injected with D-luciferin i.p., and in vivo bioluminescent imaging (BLI) was performed as described previously^21^. Next, the mice were killed in a CO_2_ gas-filled chamber, and the weight of the excised tumors was measured.

The animal experiments were performed in accordance with the institutional guidelines of the Institute of Laboratory Animal Resources (1996) and of Yeungnam University for the care and use of laboratory animals (2009).

### Statistical analysis

The results are presented as the mean ± S.E.M. and were analyzed using one-way ANOVA followed by the Newman–Keuls comparison method using the GraphPad Prism software (version 5.0) (San Diego, CA, USA). *P* values less than 0.05 were considered statistically significant.

## Results

### Synergistic effect of CTSS and MMP-9 in MDA-MB-231 human TNBC cell invasion

**Fig. 1:**
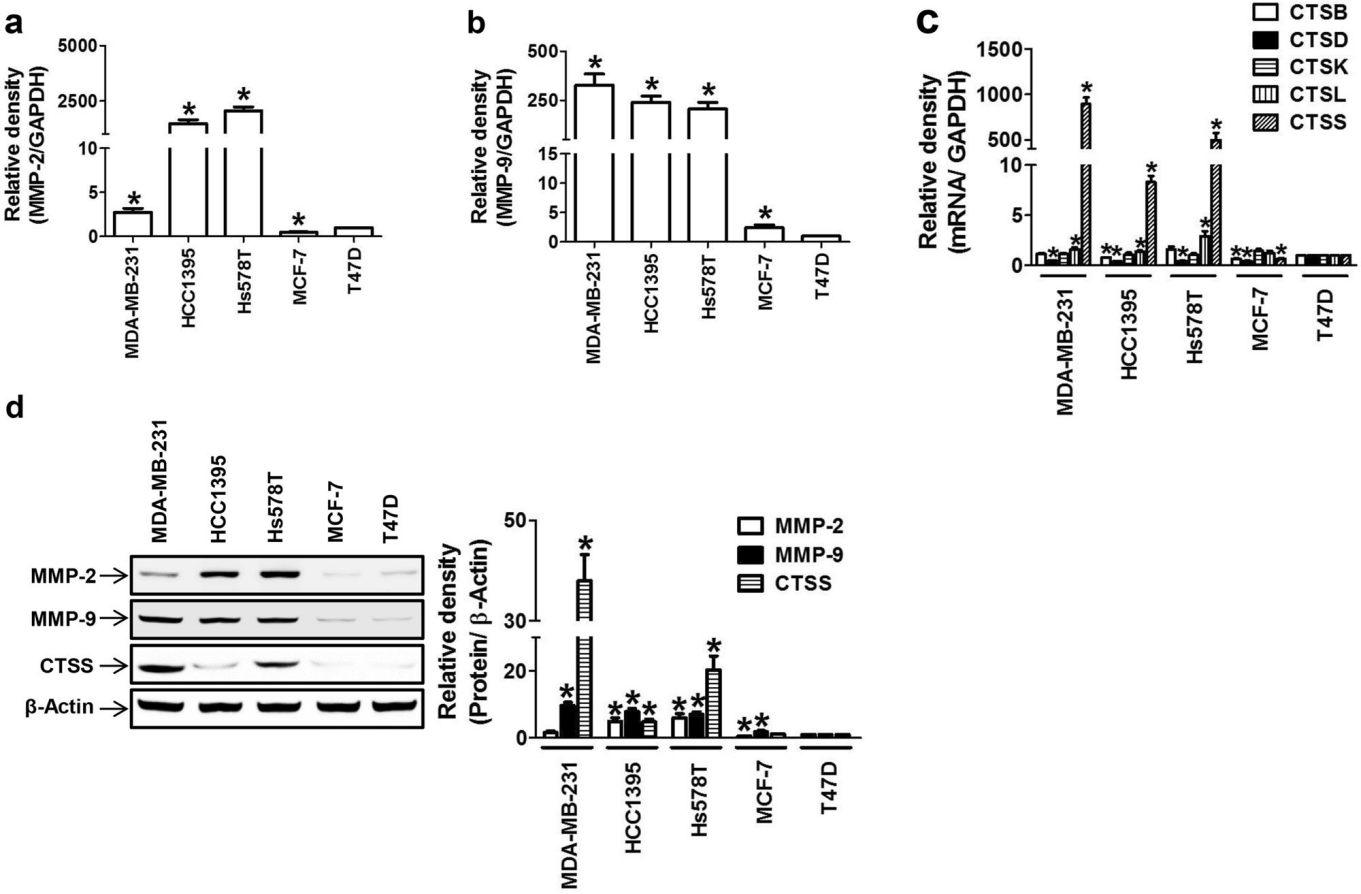
Relative expression levels of cathepsins and gelatinases in TNBC cells. **a–d** Basal mRNA expression of *MMP*-2 (**a**), *MMP*-9 (**b**), or CTSs (**c**) and their protein expression (**d**) in multiple breast cancer cell lines (MDA-MB-231, HCC-1395, Hs578T, MCF-7, and T47D). The bar-diagram represents the quantitation of the gene expression, *P < 0.05 compared to T47D cells

Based on previous reports that MMPs^33,34^ and cathepsins^14,35–38^ are highly expressed in breast tumors, we investigated the relative contribution of the proteases in breast cancer cell invasion and metastasis in multiple breast cancer cell lines. Unlike hormone-responsive breast cancer cells (MCF-7 and T47D), TNBC cells (MDA-MB-231, HCC-1973, and Hs578T) expressed higher levels of both MMP-2 (Fig. 1a, d) and MMP-9 (Fig. 1b, d). As accumulating evidence suggests the prognostic role of MMP-9 in breast cancer, the TNBC cells highly expressed MMP-9, whereas MMP-2 up-regulation was not consistent in various TNBC cell lines (Fig. 1a). In the case of cathepsins, CTSB, CTSD, CTSK, CTSL, and CTSS were expressed in all breast cancer cells; however, the mRNA (Fig. 1c) and protein (Fig. 1d) expression of CTSS was much higher in the TNBC cell lines compared to the hormone-responsive cell lines. The degree of difference in CTSS protein expression between the TNBC and hormone-responsive cell lines was much greater than that for the expression of MMP-2 or MMP-9 (Fig. 1d).

**Fig. 2:**
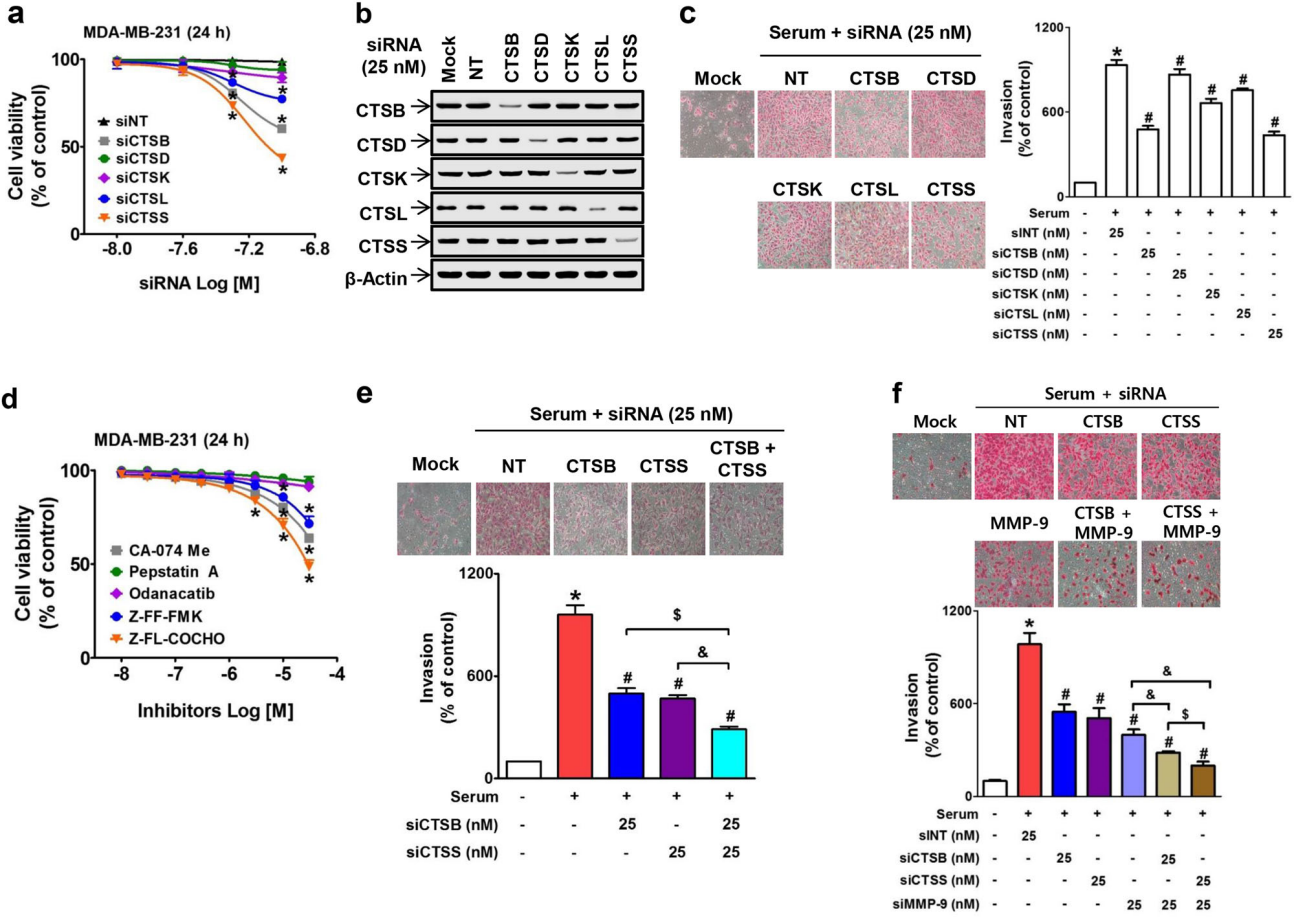
Dual roles of CTSS in viability and invasion of MDA-MB-231 breast cancer cells. **a** Cell viability of MDA-MB-231 cells transfected with different types of CTS siRNAs. **b** Transfection efficiency measurement of specific CTS siRNA (25 nM) in MDA-MB-231 cells. **c**, **e** Cells were transfected with 25 nM of the indicated siRNAs, and invasion assays were performed as described in the Methods section. The photos are representative of three independent experiments, and the bar graph indicates the mean ± S.E.M. *P < 0.05 compared to control. ^#^*P* < 0.05 compared to the serum-treated group. ^$^*P* < 0.05 compared to CTSB siRNA-transfected group. ^&^*P* < 0.05 compared to the CTSS siRNA-transfected group. **d** Cell viability of MDA-MB-231 cells treated with specific CTS inhibitors. **f** MDA-MB-231 cells were treated with siRNAs of each CTS or MMP-9, and invasion assays were performed. The bar-diagram represents the quantitation of data. *P  < 0.05 compared to the control. ^#^*P* < 0.05 compared to the serum-treated group. ^&^*P* < 0.05 compared to the MMP-9-siRNA-treated group. ^$^*P* < 0.05 compared to the CTSB-siRNA- and MMP-9-siRNA-treated group

Although CTSS expression was much higher than the expression of other cathepsins in TNBC cells, previous studies have shown that CTSB or CTSL regulates breast cancer invasion^39,40^. To investigate which cathepsin plays the most important role in highly metastatic MDA-MB-231 TNBC cell invasion, we examined the effect of siRNA-mediated knock-down of each cathepsin on the invasion ability of MDA-MB-231 cells. Transfection with CTSB, CTSL, or CTSS siRNA significantly reduced cell viability in a concentration-dependent manner, and CTSS knock-down resulted in the strongest reduction in cell viability (Fig. 2a). The non-toxic concentration (25 nM) of CTSB or CTSS siRNA transfection (Fig. 2b) significantly and more strongly inhibited MDA-MB-231 invasion than that by other cathepsins (Fig. 2c); however, it did not fully suppress the cell invasion (Fig. 2c). The role of CTSB and CTSS in breast cancer cell survival and invasion was also confirmed with the use of pharmacological inhibitors that were specific for each cathepsin (Fig. 2d, Table 2). Co-treatment with CTSB and CTSS siRNAs resulted in synergistic suppression of cell invasion (Fig. 2e). Because the inhibition of cathepsin expression or activity by a specific siRNA or inhibitor, respectively, did not completely block MDA-MB-231 cancer cell invasion, the role of MMP-9 in synergistic action with CTSB or CTSS was examined. The effect of treatment with MMP-9 siRNA alone was stronger than that with CTSB or CTSS siRNA treatment alone. The knock-down of MMP-9 in combination with the knock-down of either CTSB or CTSS resulted in synergistic suppression of MDA-MB-231 cell invasion (Fig. 2f). Similarly, the suppression of MDA-MB-231 cell invasion via co-treatment with batimastat (a pan-MMP inhibitor) and Z-FL-COCHO (a specific CTSS inhibitor) was more effective than co-treatment with batimastat and CA074-Me (a CTSB inhibitor) (Supplementary Figure S1), indicating that CTSS plays a more important role than CTSB.

**Table 2:** The effects of CTS inhibitors on the viability and serum-induced invasion of MDA-MB-231 cells

**CTS inhibitor**	**Concentration**	**Invasion (% inhibition)**
CA-074 Me (nM)	1	22.16 ± 2.58^#^
	5	38.49 ± 2.57^#^
	25	49.95 ± 1.52^#^
Pepstatin A (μM)	1	3.81 ± 0.81
	5	4.49 ± 0.97
	25	6.17 ± 0.96
Odanacatib (μM)	0.04	16.71 ± 4.91
	0.2	22.36 ± 4.57^#^
	1	25.05 ± 4.28^#^
Z-FF-FMK (μM)	0.04	3.83 ± 2.33
	0.2	7.79 ± 2.63
	1	11.34 ± 2.42^#^
Z-FL-COCHO (nM)	0.4	38.48 ± 0.92^#^
	2	50.05 ± 0.89^#^
	10	56.17 ± 0.93^#^

Values are mean ± S.E.M. of three independent experiments

^#^*P* < 0.05 compared to the serum-treated group

### Comparison of the inhibitory effects of Z-FL-COCHO, batimastat, and BJ-2302, a bicyclic pyridinol derivative (an angiogenesis inhibitor), on CTSS activity, TNBC cell invasion, and cytotoxicity

**Fig. 3:**
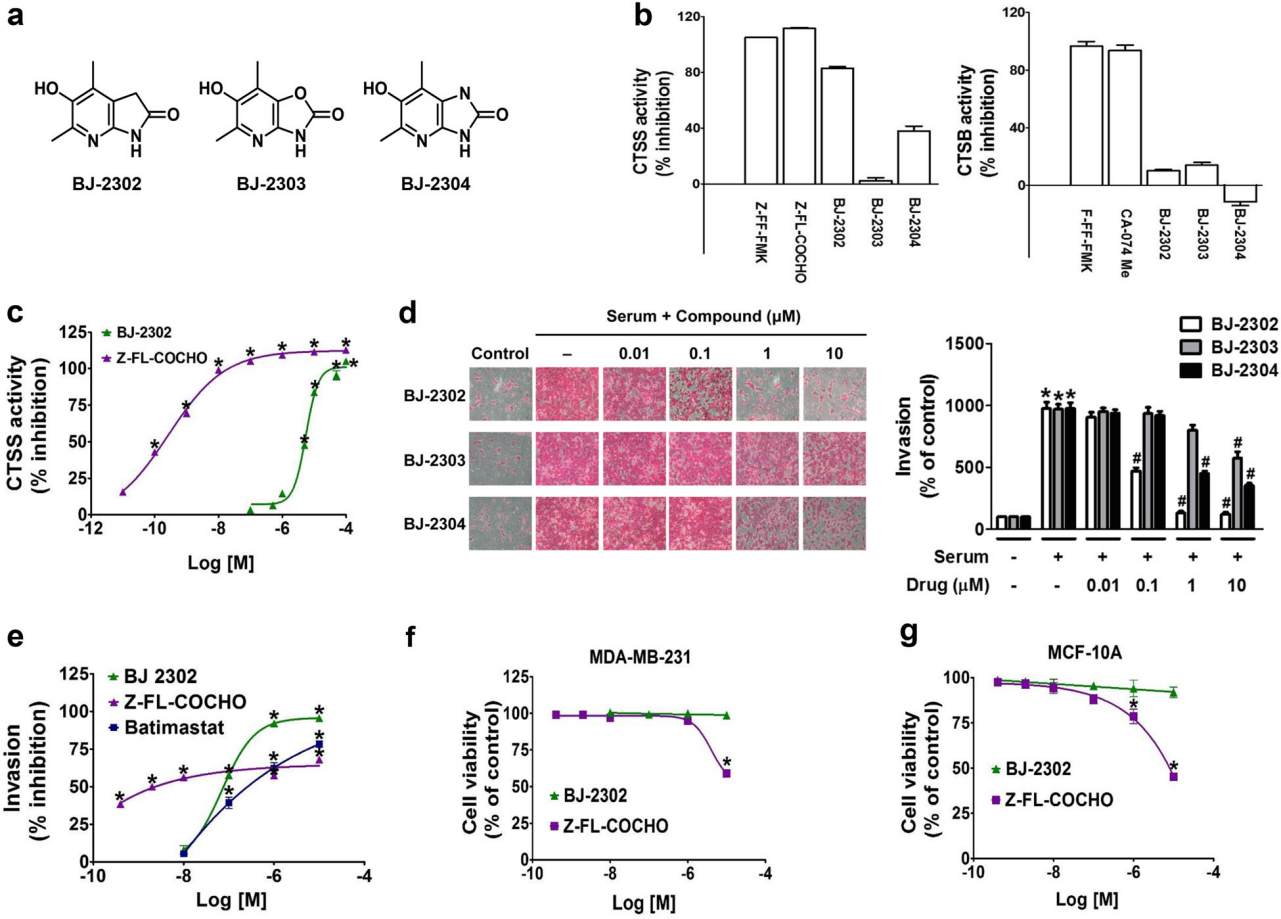
Effects of bicyclic aminopyridinol compounds on CTSS activity, invasion, and viability of MDA-MB-231 cells. **a** Chemical structure of three bicyclic aminopyridinol compounds, BJ-2302, BJ-2303, and BJ-2304. **b**, **c** Inhibitory effects of the three compounds on CTSS and CTSB activity. *P < 0.05 compared to vehicle-treated controls. **d** MDA-MB-231 cells were pretreated with the compounds, and a serum-induced invasion assay was performed. The photos are representative of three independent experiments, and the bar-diagram represents the mean ± S.E.M. *P < 0.05 compared to vehicle-treated cells. ^#^*P* < 0.05 compared to serum-treated cells. **e** Measurement of the IC_50_ of serum-induced invasion of MDA-MB-231 cells treated with Z-FL-COCHO, batimastat, or BJ-2302. *P < 0.05 compared to serum-treated cells. **f**, **g** Effect of BJ-2302 or Z-FL-COCHO on the cell viability of MDA-MB-231 breast cancer cells (**f**) and MCF-10A normal breast cells (**g**). *P < 0.05 compared to vehicle-treated cells

Angiogenesis also plays an important role in cancer progression, and CTSS is involved in angiogenesis through degrading the matrix for endothelial cell invasion and migration. Based on our previous study, which had demonstrated anti-angiogenic actions of bicyclic pyridinol compounds^40^, in the present study, we examined whether bicyclic pyridinol compounds exhibit CTSS inhibitory activity. Among the three structurally related compounds, that is, BJ-2302, BJ-2303, and BJ-2304 (Fig. 3a), BJ-2302 exhibited the strongest suppression of CTSS activity (Fig. 3b), followed by BJ-2304. In contrast, none of the compounds showed strong inhibition of CTSB activity (Fig. 3b). The IC_50_ value of BJ-2302 against CTSS activity was 5.1 µM (Fig. 3c), which was higher than that of Z-FL-COCHO (0.19 nM), a potent CTSS inhibitor (positive control). The degree of CTSS inhibition by these three compounds corresponded to their inhibitory effect on serum-induced invasion of MDA-MB-231 cells (Fig. 3d). Compared to Z-FL-COCHO and batimastat (pan MMP inhibitor), BJ-2302 inhibited MDA-MB-231 cell invasion in a dose-dependent manner (Fig. 3e), and the IC_50_ values of BJ-2302, Z-FL-COCHO, and batimastat on cell invasion were 0.08, 0.0049, and 0.26 µM, respectively. However, the maximum inhibitory effect of BJ-2302 was more potent than that of Z-FL-COCHO or batimastat, as evidenced from the IC_90_ values of BJ-2302, Z-FL-COCHO, and batimastat against MDA-MB-231 cell invasion (0.71, >10, and >10 μM, respectively) (Fig. 3e). Despite such strong anti-invasive activity, BJ-2302 did not induce cytotoxicity in the MDA-MB-231 cancer cells, whereas Z-FL-COCHO was cytotoxic to MDA-MB-231 at a concentration of 10 μM (Fig. 3f). Further examination of the cytotoxic profile using MCF-10A, a normal breast epithelial cell line, revealed that BJ-2302 was not cytotoxic, whereas Z-FL-COCHO induced cytotoxicity in a concentration-dependent manner (Fig. 3g).

### Anti-metastatic effect of BJ-2302, a non-cytotoxic CTSS inhibitor, in a CAM xenograft model inoculated with MDA-MB-231-luc2-tdTomato cancer cells

**Fig. 4:**
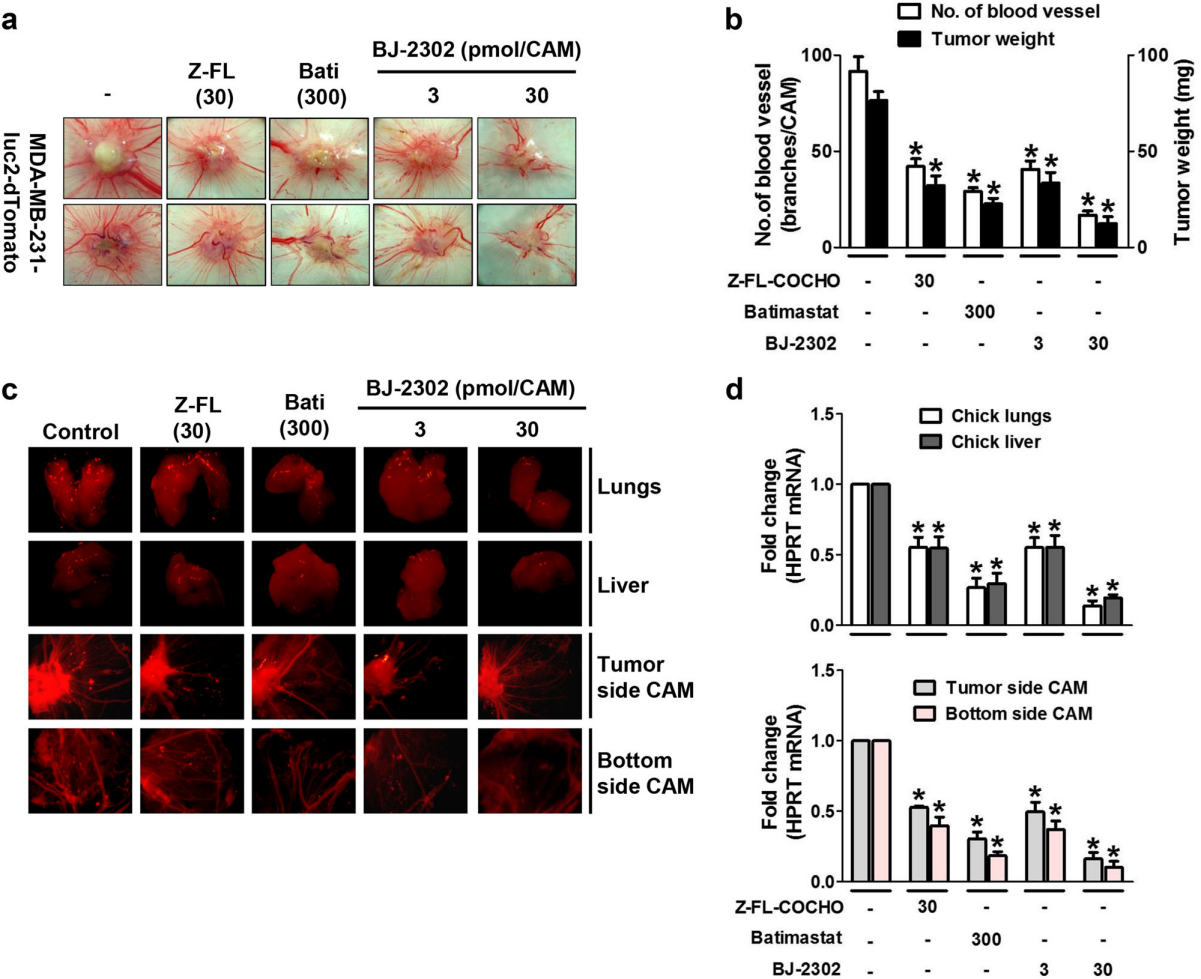
Anti-metastatic effect of BJ-2302, a non-cytotoxic CTSS inhibitor, in an MDA-MB-231-luc-2-tdTomato cell-xenografted CAM model. MDA-MB-231-luc-2-tdTomato cancer cells were inoculated at the top of the CAM in the presence of indicated drugs. **a–d** On the 5th day of drug treatment, tumor growth was visualized with images captured under the stereomicroscope (**a**), and the number of blood vessels within the tumor area and tumor weights (**b**) were measured. **c**, **d** Images of the developing lungs, liver, lower CAM, and upper CAM were captured for the detection of red-fluorescence-labeled cancer cells (**c**), and the tissues were examined for *HPRT* mRNA in the chick lungs and liver (upper bar) and the upper and lower parts of the CAM (lower bar) (**d**). The bar-diagram represents quantitative data. *P < 0.05 compared to vehicle-treated groups

Next, we examined the in vivo anti-metastatic activity of BJ-2302 using a CAM xenograft model. In CAMs inoculated with MDA-MB-231-luc2-tdTomato cancer cells, the effects of BJ-2302 on tumor growth, tumor-induced angiogenesis, and cancer cell metastasis were analyzed. BJ-2302 significantly suppressed tumor growth and tumor-induced angiogenesis in a concentration-dependent manner and was more effective than Z-FL-COCHO or batimastat (Fig. 4a, b). Metastasis of MDA-MB-231-luc2-tdTomato cells was assessed by identifying red-fluorescence-labeled cancer cells in developing organs underneath the CAM, such as the lungs and liver, and in the bottom CAM, which was the opposite site of the cancer cell inoculation. Compared to Z-FL-COCHO or batimastat, BJ-2302 significantly suppressed the metastasis of MDA-MB-231-luc2-tdTomato cells to the lungs, liver, and the bottom CAM (Fig. 4c). The degree of metastasis was quantitated via measuring the expression of the human housekeeping gene *HPRT*. BJ-2302 suppressed *HPRT* mRNA expression in the liver and lungs of the developing chick embryo (Fig. 4d, upper), as well as in the upper and bottom CAMs (Fig. 4d, lower). This activity of BJ-2302 was more potent than that of Z-FL-COCHO or batimastat.

### Inhibition of Src abrogated CTSS and MMP-9 expression via dual suppression of the PI3K/Akt and Ras/Raf/ERK pathways

**Fig. 5:**
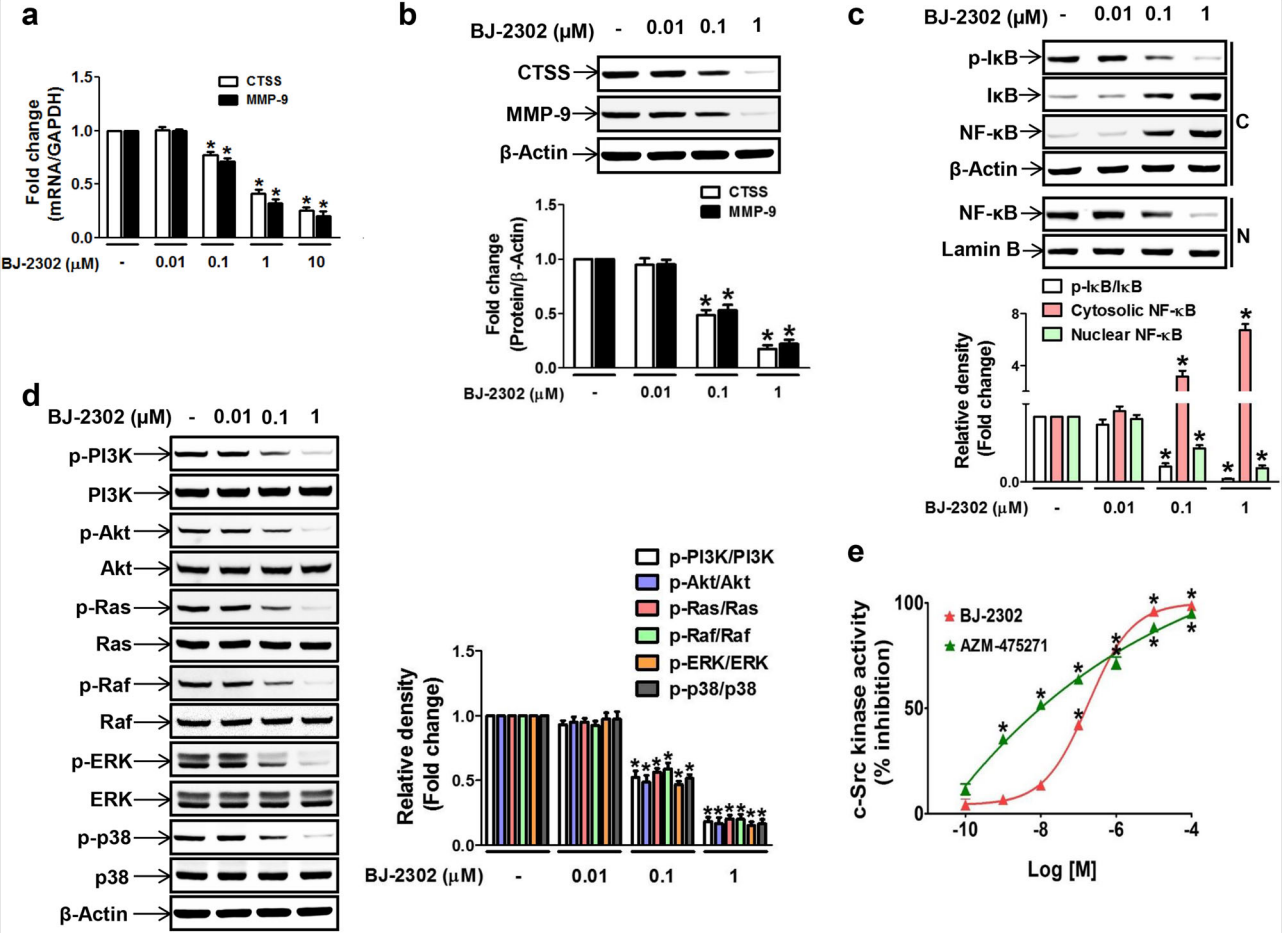
Down-regulation of CTSS and MMP-9 expression by BJ-2302 via inhibition of Src and its down-stream signaling pathways, PI3K/Akt and Ras/Raf/ERK. **a**, **b** MDA-MB-231 cells were treated with the indicated concentrations of BJ-2302 and were examined for the mRNA (**a**) and protein levels (**b**) of CTSS and MMP-9. The bands are representative of three independent experiments, and the bar-diagram represents the mean ± S.E.M. *P < 0.05 compared to vehicle-treated cells. **c**, **d** Effect of BJ-2302 on NF-κB nuclear translocation (**c**) and upstream molecules (**d**) related to the regulation of CTSS and MMP-9 expression. The bar-diagram represents quantitative data. *P < 0.05 compared to vehicle-treated cells. **e** Comparison of the inhibitory effect of BJ-2302 with AZM-475271 on Src kinase activity. *P < 0.05 compared to vehicle-treated control

The anti-invasive and anti-metastatic activity of BJ-2302 was more effective than that of Z-FL-COCHO, indicating that the mode of action of BJ-2302 was not confined to the inhibition of CTSS activity. To investigate this mechanism, we examined whether the anti-invasive and anti-metastatic activity of BJ-2302 was associated with its inhibitory action on the gene expression of proteases. BJ-2302 induced a decrease in the basal levels of mRNA (Fig. 5a) and protein expression (Fig. 5b) of both CTSS and MMP-9. Such concurrent inhibition of both CTSS and MMP-9 further indicates that BJ-2302 might act on a common signaling molecule that is involved in the regulation of both CTSS and MMP-9 expression. Previous reports have shown that the PI3K/Akt and Ras/Raf/ERK pathways merged at NF-κB activation, which regulates CTSS and MMP-9 expression^14,41^. In MDA-MB-231 cells, BJ-2302 significantly suppressed nuclear translocation of NF-κB (Fig. 5c). In addition, BJ-2302 inhibited the phosphorylation of all upstream signaling molecules (PI3K, Akt, Ras, Raf, ERK, and p38) (Fig. 5d). Moreover, because Src is an upstream molecule of both the PI3K/Akt and Ras/Raf/ERK pathways^21^, we examined the direct inhibitory effect of BJ-2302 on Src activity. BJ-2302 inhibited Src kinase activity more strongly than did AZM-475271, a known Src inhibitor. The IC_90_ values of BJ-2302 and AZM-475271 were 3.2 and 27.3 µM, respectively (Fig. 5e).

**Fig. 6:**
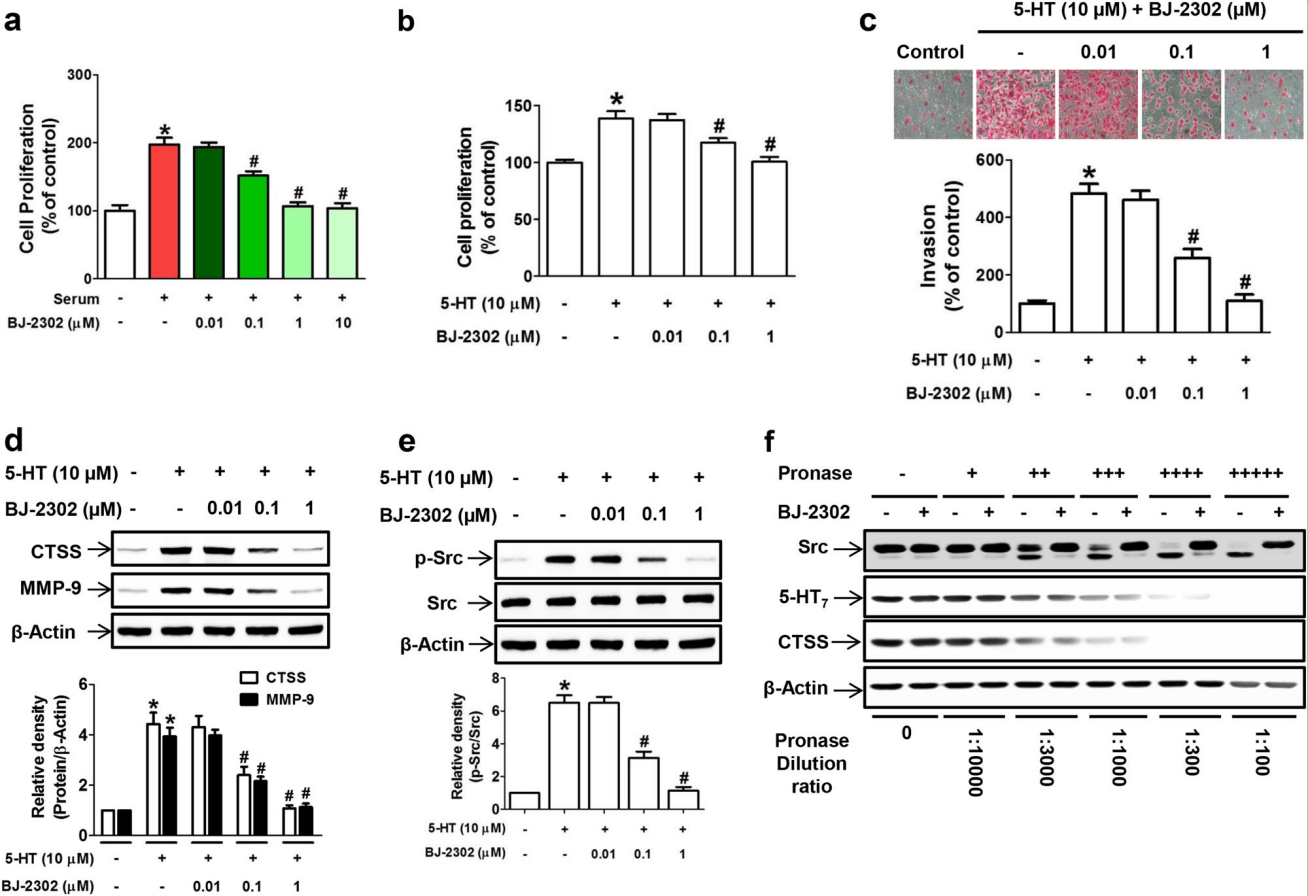
Suppression of TNBC cell progression by BJ-2302. **a**, **b** MDA-MB-231 cell proliferation induced by serum (**a**) or 5-HT (**b**) was measured in the presence of BJ-2302. **c** 5-HT-induced MDA-MB-231 cell invasion, **d**, **e** 5-HT-induced protein expression of CTSS and MMP-9 (**d**) and phosphorylation of Src (**e**) were suppressed by BJ-2302. The bar graphs represent the mean ± S.E.M. from three independent experiments *P < 0.05 compared to vehicle-treated cells. ^#^*P* < 0.05 compared to serum- or 5-HT-treated cells. **f** DARTS assay showing direct binding of BJ-2302 to Src but not to the 5-HT_7_ receptor or CTSS protein. The bands are representative of three independent experiments

Because the PI3K/Akt and Ras/Raf/ERK signaling pathways are involved in cancer cell proliferation, which is the malignant process after invasion^43–45^, the anti-proliferative activity of BJ-2302 was investigated. BJ-2302 inhibited serum-induced MDA-MB-231 cell proliferation in a concentration-dependent manner and exhibited a complete suppression at a concentration of 1 μM (Fig. 6a). Because TNBC invasion and proliferation are also regulated via the 5-HT_7_ receptor-linked signaling pathway^21^, in the present study, we also investigated the inhibitory effect of BJ-2302 on 5-HT-stimulated MDA-MB-231 TNBC cell proliferation and invasion. BJ-2302 inhibited 5-HT-induced cell proliferation (Fig. 6b), invasion (Fig. 6c), and the protein expression of CTSS and MMP-9 (Fig. 6d) in a concentration-dependent manner. Similarly, 5-HT-induced phosphorylation of Src protein in MDA-MB-231 cells was significantly suppressed by BJ-2302 in a concentration-dependent manner (Fig. 6e). To further determine the action of BJ-2302 as a Src inhibitor, a drug affinity responsive target stability (DARTS) assay was performed, which relies on the principle that the protein becomes stabilized upon binding or interacting with a small molecule and becomes resistant to protease action. In the presence of BJ-2302, Src protein was not digested by pronase, while 5-HT_7_ receptor protein was degraded by pronase (Fig. 6f). Moreover, although BJ-2302 showed a weak CTSS inhibitory activity (Fig. 3c), the presence of 1 µM BJ-2302 did not protect against CTSS protein digestion by pronase. The results implicate that Src, but not 5-HT_7_ or CTSS, was the high-affinity target for BJ-2302 action.

### Anti-tumor activity of BJ-2302 in a mouse xenograft model with MDA-MB-231 breast cancer cells

**Fig. 7:**
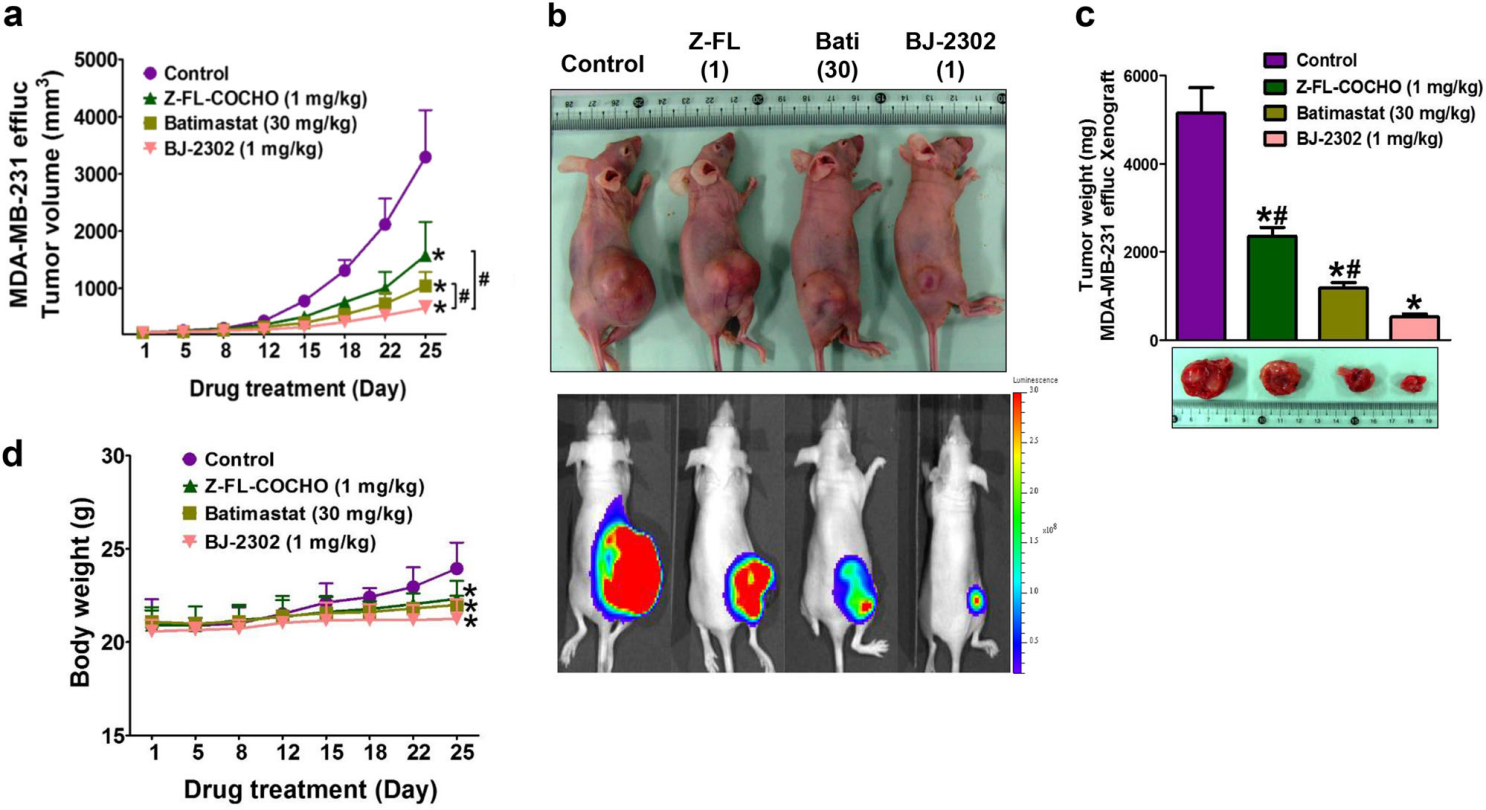
BJ-2302 inhibited tumor growth in MDA-MB-231 tumor xenografted models. Z-FL-COCHO (1 mg/kg), batimastat (30 mg/kg), or BJ-2302 (1 mg/kg) was administered intraperitoneally to mice xenografted with MDA-MB-231-effLuc cells. **a**, **d** The mean tumor volume (**a**) and mouse body weight (**d**) were measured during the drug treatment period. **b**, **c** On the 25th day of drug treatment, BLI was performed (**b**), and excised tumor weights were measured (**c**). Each group contained six mice. The bar-diagram represents quantitative data. *P < 0.05 compared to vehicle-treated mice. ^#^*P* < 0.05 compared to BJ-2302-treated mice

The anti-cancer activity of BJ-2302 was further confirmed in an MDA-MB-231 cancer xenograft mouse model. BJ-2302 (1 mg/kg) significantly suppressed tumor growth (Fig. 7a). This suppression was greater than that achieved using Z-FL-COCHO (1 mg/kg) or batimastat (30 mg/kg) (Fig. 7a). In vivo optical imaging revealed that the BLI signals emitted by the tumors (Fig. 7b) and the excised tumor weight (Fig. 7c) were much lower in the BJ-2302-administered mice than in the Z-FL-COCHO- or batimastat-treated mice. However, the BJ-2302-, Z-FL-COCHO-, or batimastat-treated mice did not exhibit any progressive body weight loss (Fig. 7d).

## Discussion

Previous studies have reported the increased expression of different types of cathepsins such as CTSB, CTSD, CTSK, CTSL, and CTSS in breast cancer^46–50^. Although CTSD, an aspartyl protease, is overexpressed and secreted by breast carcinomas, we demonstrated that cysteine proteases (CTSB, CTSK, CTSL, and CTSS) rather than the aspartyl proteases participated in TNBC invasion through blocking or inhibiting them individually via siRNA transfection and specific inhibitors. Among cysteine cathepsins, CTSB and CTSS were the major players in the regulation of invasion in the highly metastatic TNBC cell line MDA-MB-231. At the same time, suppression of CTSB or CTSS by either specific small-molecule inhibitors or siRNA decreased cell viability. This result is consistent with previous reports that cathepsins are also involved in the regulation of autophagy, which indicates that cathepsins might play a dual role in promoting cancer cell survival and also inducing cell death^51,52^. However, our current study showed that CTSS had more of an impact than CTSB on cell survival. Additionally, compared to other cathepsins, the special characteristics of CTSS, such as exhibiting limited tissue distribution and stability at both an acidic and neutral pH^16,53^, have made CTSS a promising target for the management of TNBC invasion. However, our present study showed that the inhibition of CTS expression or activity via a specific siRNA or inhibitor, respectively, did not block the invasion completely; this result suggested a critical role of other proteases such as MMP-9 in TNBC invasion. Although previous studies have suggested a close association of MMP-9 with triple negativity of breast cancer, metastasis, and the poor prognosis of TNBC^5,6^, the current study demonstrated that similar to CTSS, the suppression of MMP-9 alone was not enough to block the TNBC invasion. Instead, the combined inhibition of both MMP-9 and CTSS showed a strong synergistic effect on the suppression of MDA-MB-231 cell invasion. These results suggest that any drug that blocks TNBC progression should possess dual inhibitory activity against both CTSS and MMP-9, and this might be the criteria for anti-invasive, anti-metastatic drug development against TNBC.

Several lines of evidence implicate the putative role of various endoproteases (such as MMP-9 and CTSS) in the process of angiogenesis^54–58^ through the release and activation of growth factors (to enhance neovascularization) and degradation of ECM components (to promote endothelial cell invasion and migration). Based on these reports, bicyclic pyridinol compounds that have potent anti-angiogenic activity in vivo^59^ were screened for CTSS inhibition. Structurally similar bicyclic pyridinol derivatives (BJ-2302, BJ-2303, and BJ-2304) showed differential inhibitory activity against CTSS and MDA-MB-231 cell invasion. BJ-2302, which showed the highest inhibition of CTSS activity, suppressed the expression of both CTSS and MMP-9 and the invasion of MDA-MB-231 cells. Such in vitro anti-invasive action of BJ-2302 was confirmed in an in vivo metastasis model using a CAM inoculated with MDA-MB-231 luc2-tdTomato cells. The inhibitory effects of BJ-2302 were much stronger than those of Z-FL-COCHO (specific CTSS inhibitor) and batimastat (pan-MMP inhibitor). A previous report had shown that there was no correlation between the CTSS enzyme inhibitory activity of novel compounds (α-ketoamide) and the degree of inhibition of migration in CL1-3 cells that express considerably higher CTSS levels than MDA-MB-231 cells^60^. Taking this result into consideration, our current study showed a close correlation between the in vitro and in vivo results, which demonstrated that our current approach to discovering anti-invasive drug candidates is appropriate.

**Fig. 8:**
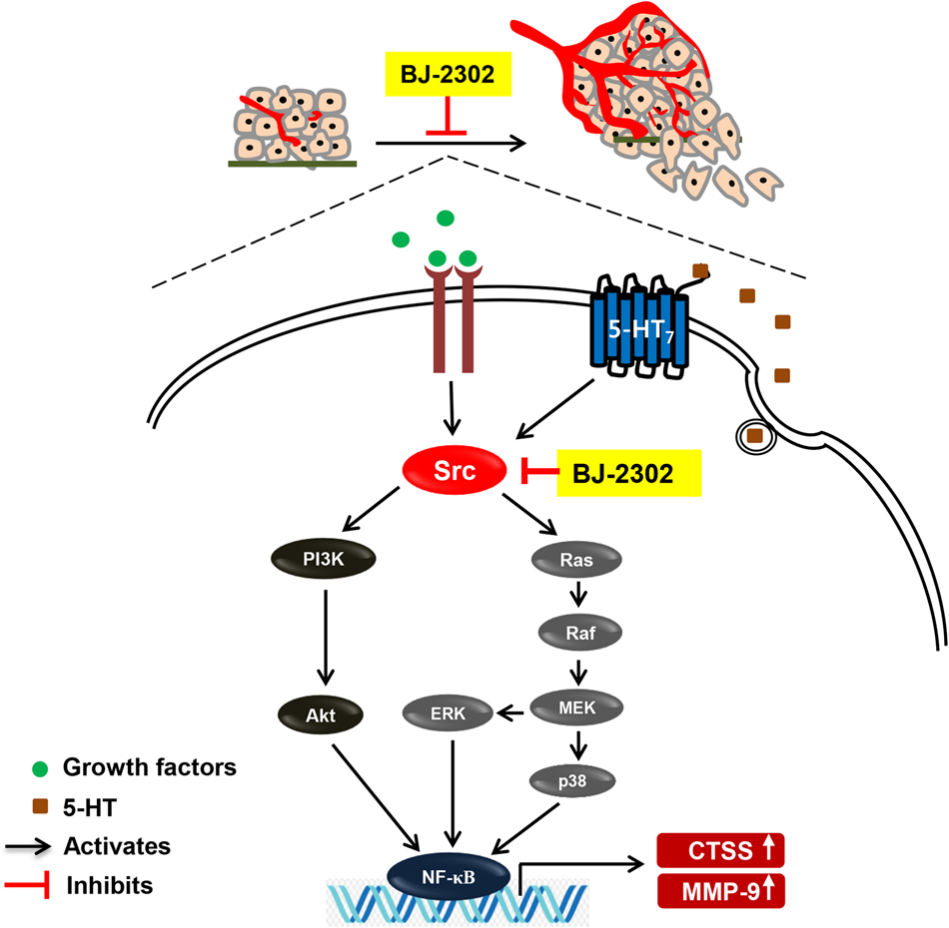
Proposed signaling mechanism for the anti-cancer effect of BJ-2302, a Src inhibitor, in MDA-MB-231 human TNBC cells. The anti-metastatic, anti-tumor effect of BJ-2302 was mediated by the down-regulation of CTSS and MMP-9 protein expression via suppression of Src-mediated dual activation of the PI3K/Akt and Ras/Raf/ERK pathways

In addition to invasion and metastasis, BJ-2302 also inhibited cell proliferation in vitro and MDA-MB-231 tumor growth in vivo. BJ-2302 also caused the inhibition of the signaling pathways PI3K/Akt and Ras/Raf/ERK, both of which are regulated by Src in MDA-MB-231 cells. Src kinase is activated by various GFRs^61^ and G-protein coupled receptors such as 5-HT_7_ receptors, a highly overexpressed 5-HT receptor in the cells^21^. BJ-2302 inhibiting Src kinase blocked not only serum-induced but also 5-HT-induced TNBC progression via inhibiting proliferation, invasion, and protein expression of CTSS and MMP-9. This anti-tumor activity of BJ-2302 was confirmed in a xenograft mouse tumor model. Our current study also demonstrated that although their effects were much weaker than BJ-2302, CTSS and MMP inhibitors (Z-FL-COCHO and batimastat) suppressed tumor growth in vivo. This result is consistent with previous reports that proteases such as MMP and cathepsins play an important role in cancer cell proliferation^54,57,58^. Consistent with our previous study, which showed that Src is a mediator of 5-HT_7_-receptor-induced TNBC progression^21^, elevated Src activity is correlated with reduced survival time in TNBC patients^62^. In our current study, BJ-2302 inhibited Src kinase activity more efficiently than AZM-475271, a Src kinase inhibitor exhibiting potent anti-cancer and anti-metastatic activities^63^. In conjunction with those reports and the results from the current study regarding BJ-2302, it may be concluded that the suppression of Src kinase and the resultant down-regulation of CTSS and MMP-9 expression inhibited proliferation, invasion, and metastasis of MDA-MB-231 cells (Fig. 8).

Importantly, BJ-2302 was not cytotoxic to the MCF-10A normal human breast cell line, which indicates the cancer-specific action of BJ-2302. This result enhances the potential of BJ-2302 as an anti-metastatic, anti-cancer drug. Thus, the inhibition of Src kinase activity by BJ-2302 constitutes a novel approach to cancer therapy against drug-resistant and highly metastatic TNBC growth and metastasis.

## Electronic supplementary material

Supplementary Figure S1
