# Supplementary material for: Down-regulation of cathepsin S and matrix metalloproteinase-9 via Src, a non-receptor tyrosine kinase, suppresses triple-negative breast cancer growth and metastasis
Source: Exp Mol Med. 2018 Sep 5;50(9):118. doi: 10.1038/s12276-018-0135-9 (PMC6123788; doi:10.1038/s12276-018-0135-9)
Supplement: Supplementary file 1 — Supplementary Figure S1 [file 12276_2018_135_MOESM1_ESM.pptx]

## Slide 1
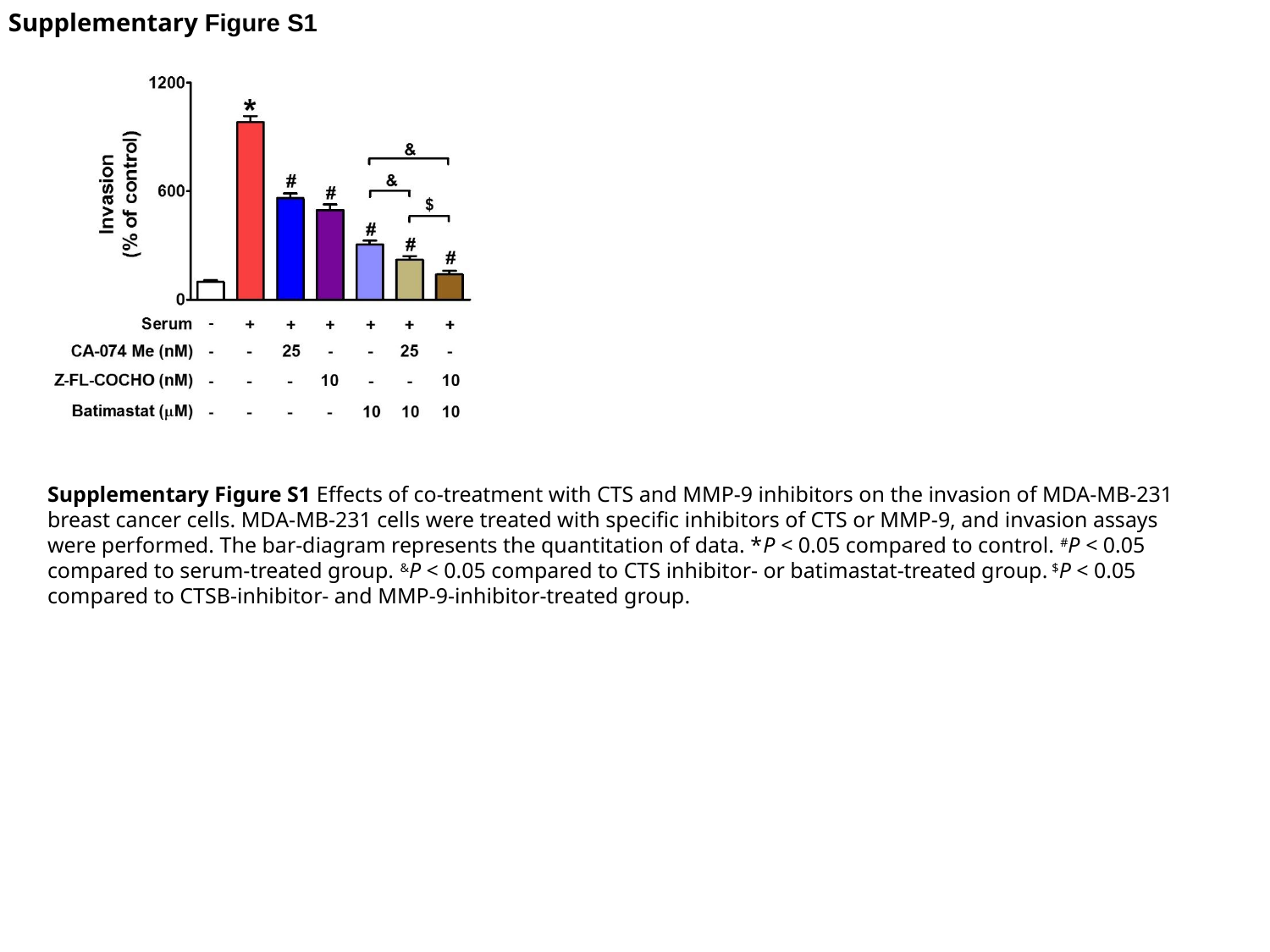

Supplementary Figure S1
Supplementary Figure S1 Effects of co-treatment with CTS and MMP-9 inhibitors on the invasion of MDA-MB-231 breast cancer cells. MDA-MB-231 cells were treated with specific inhibitors of CTS or MMP-9, and invasion assays were performed. The bar-diagram represents the quantitation of data. *P < 0.05 compared to control. #P < 0.05 compared to serum-treated group. &P < 0.05 compared to CTS inhibitor- or batimastat-treated group. $P < 0.05 compared to CTSB-inhibitor- and MMP-9-inhibitor-treated group.
